# Translational Hemorrhagic Stroke: Physiology, Pharmaceutical Drugs, and Management

**DOI:** 10.1155/2017/3248010

**Published:** 2017-11-15

**Authors:** John H. Zhang, Mario Zucarello, Serge Marbacher, Gang Chen, Sheng Chen

**Affiliations:** ^1^Departments of Neurosurgery, Physiology, and Anesthesiology, Loma Linda University School of Medicine, Loma Linda, CA 92354, USA; ^2^Department of Neurosurgery, University of Cincinnati, Cincinnati, OH 45219, USA; ^3^Department of Neurosurgery, Kantonsspital Aarau, Tellstrasse, 5001 Aarau, Switzerland; ^4^Department of Neurosurgery, Soochow University, Jiangsu, China; ^5^Department of Neurosurgery, Second Affiliated Hospital, School of Medicine, Zhejiang University, Zhejiang, China

We are pleased to announce the publication of a special issue on major research challenges and achievements in the physiology, pharmaceutical drug therapy, and management of “hemorrhage stroke.” The call for papers on this topic resulted in a significant amount of interest from researchers all over the world. A total of 27 articles were submitted for review. Our editorial team selected 14 basic and clinical search articles for publication and rejected the remaining 13 (an acceptance rate of 52%). Half of the accepted manuscripts are review articles which summarize current literature on diseases such as cervical artery dissection, hydrocephalus after subarachnoid hemorrhage, intracranial atherosclerosis, or hypertensive intracerebral hemorrhage. We are confident that this special issue will advance the understanding and research of the broad range of disorders referred to as “hemorrhagic stroke.”

